# Rare, Intense, Big fires dominate the global tropics under drier conditions

**DOI:** 10.1038/s41598-017-14654-9

**Published:** 2017-10-30

**Authors:** Stijn Hantson, Marten Scheffer, Salvador Pueyo, Chi Xu, Gitta Lasslop, Egbert H. van Nes, Milena Holmgren, John Mendelsohn

**Affiliations:** 1 Karlsruhe Institute of Technology, Institute of Meteorology and Climate research, Atmospheric Environmental Research, 82467 Garmisch-Partenkirchen, Germany; 2 Environmental Sciences Department, Wageningen University, Wageningen, The Netherlands; 3 Dept. de Biologia Evolutiva, Ecologia i Medi Ambient, Universitat de Barcelona, Av. Diagonal 645, 08028 Barcelona, Catalonia Spain; 4 School of Life Sciences, Nanjing University, 210093 Nanjing, China; 5 Max Planck Institute for Meteorology, Fire in the Earth System, 20146 Hamburg, Germany; 6 Resource Ecology Group, Wageningen University, Wageningen, The Netherlands; 7 Research and Information Services of Namibia (RAISON), PO Box, 1405 Windhoek, Namibia

## Abstract

Wildfires burn large parts of the tropics every year, shaping ecosystem structure and functioning. Yet the complex interplay between climate, vegetation and human factors that drives fire dynamics is still poorly understood. Here we show that on all continents, except Australia, tropical fire regimes change drastically as mean annual precipitation falls below 550 mm. While the frequency of fires decreases below this threshold, the size and intensity of wildfires rise sharply. This transition to a regime of Rare-Intense-Big fires (RIB-fires) corresponds to the relative disappearance of trees from the landscape. Most dry regions on the globe are projected to become substantially drier under global warming. Our findings suggest a global zone where this drying may have important implications for fire risks to society and ecosystem functioning.

## Introduction

Forests, savannas and treeless vegetation represent alternative states over a precipitation gradient in the tropics^1,2^. A bistability of tropical forest versus savanna is thought to be mostly maintained by fire and herbivores driving vegetation towards an open savanna condition despite high rainfall levels^3–5^. However, it is unclear how vegetation, climate and fire interact at the lower end of the precipitation gradient, between treeless conditions and savannas. It has been suggested that precipitation determines tree cover for areas with mean annual precipitation <650–1000 mm^3,6^, resulting in a gradual increase in (maximum) tree cover over a precipitation gradient. Instead, remotely sensed data of global tree cover indicate that there is a distinct ‘treeless state’ with little or no trees, of which the likelihood becomes higher at low precipitation^1^. While fire frequency is widely considered the main factor in maintaining savanna vegetation in moist regions^3–5^, in the case of dry areas it is often assumed that fires do not drive tree dynamics because of their infrequent occurrence^7,8^. However, ample evidence indicates that in these dry areas large scaled tree establishment occurs after wet years and that fire strongly limits sapling recruitment^9,10^ and relative high tree cover do occur at a local scale^6^, indicating that climate alone does not seem to be able to explain the overall very low tree cover below ~550 mm precipitation. Here we explore whether this marked shift between treeless and savanna might be related to a change in fire regime over the global tropics (17° N − 35° S).

## Results and Discussion

Although wildland fires are generally characterised by burned area extent, many more factors describe a fire regime (e.g. intensity, size, return interval) and determine the resulting impact of fire on the environment^11,12^. The spatial characteristics of fire occurrence and fire-vegetation feedbacks are often explained by Self-Organized Criticality (SOC) theory^13^. In these SOC “forest fire” models, the interplay between regrowth of vegetation and the action of fire makes the system evolve to a scale invariant regime, where the size distribution of fires follows a power law^14,15^:

1 $$f({A}_{F})=\alpha {A}_{F}^{-\beta }$$

where $f({A}_{F})$ is the frequency density of fires with size *A*_*F*_, and *α* and −*β* are empirical parameters. We use the Slope of the Fire Size Distribution (SFSD), indicated by −*β*, to characterise the spatial properties of wildfires. The value of SFSD thus describes the relative frequencies of fires of different sizes. A SFSD of zero indicates that all sizes of fire are equally frequent, whereas lower values of SFSD indicate that smaller fires are more frequent than larger fires. The power law is fitted to individual fire sizes computed from the MODIS burned area product MCD45^16^ (see Supplementary Methods and Fig. S1).

**Figure 1:**
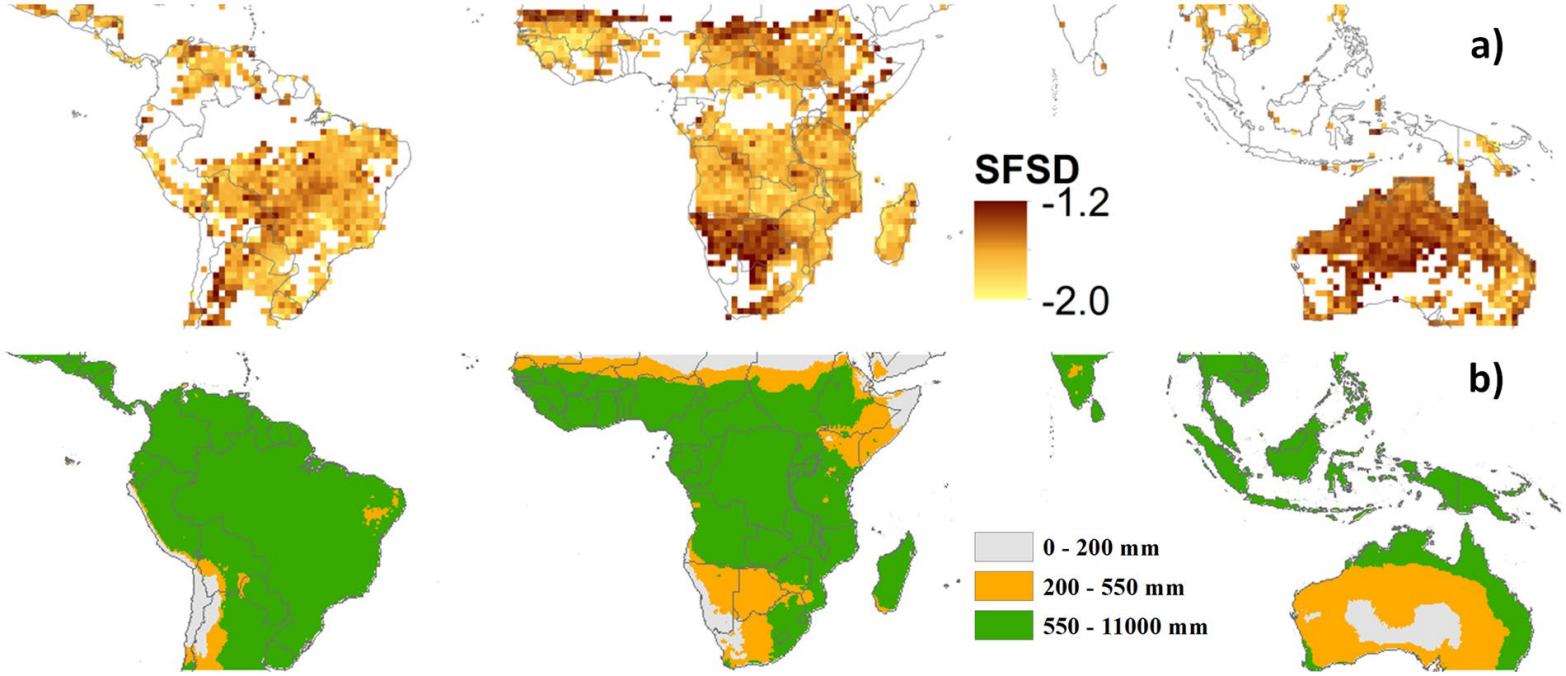
Fire size distribution (SFSD) for the tropical region (**a**) excluding areas with >25% agricultural cover. The values for SFSD are expressed as −$\beta$ exponent of the power law (Eq. 1), with darker colours having a higher frequency of large fires. Based on the 550 mm mean annual precipitation threshold for current climate the estimated spatial distribution of the fire regime is presented (**b**). This figure was produced using ArcMAP 10.2 http://desktop.arcgis.com/de/arcmap/.

The SFSD shows a clear global pattern (Fig. 1). The tropical savannas, north and south of the central African rainforest and large parts of South America, show low values of SFSD, indicating that these regions are dominated by frequent small fires. By contrast, high values of the SFSD in southern Africa, the Sahel and Australia, indicate that these drier regions are characterised by a higher frequency of larger fires.

**Figure 2:**
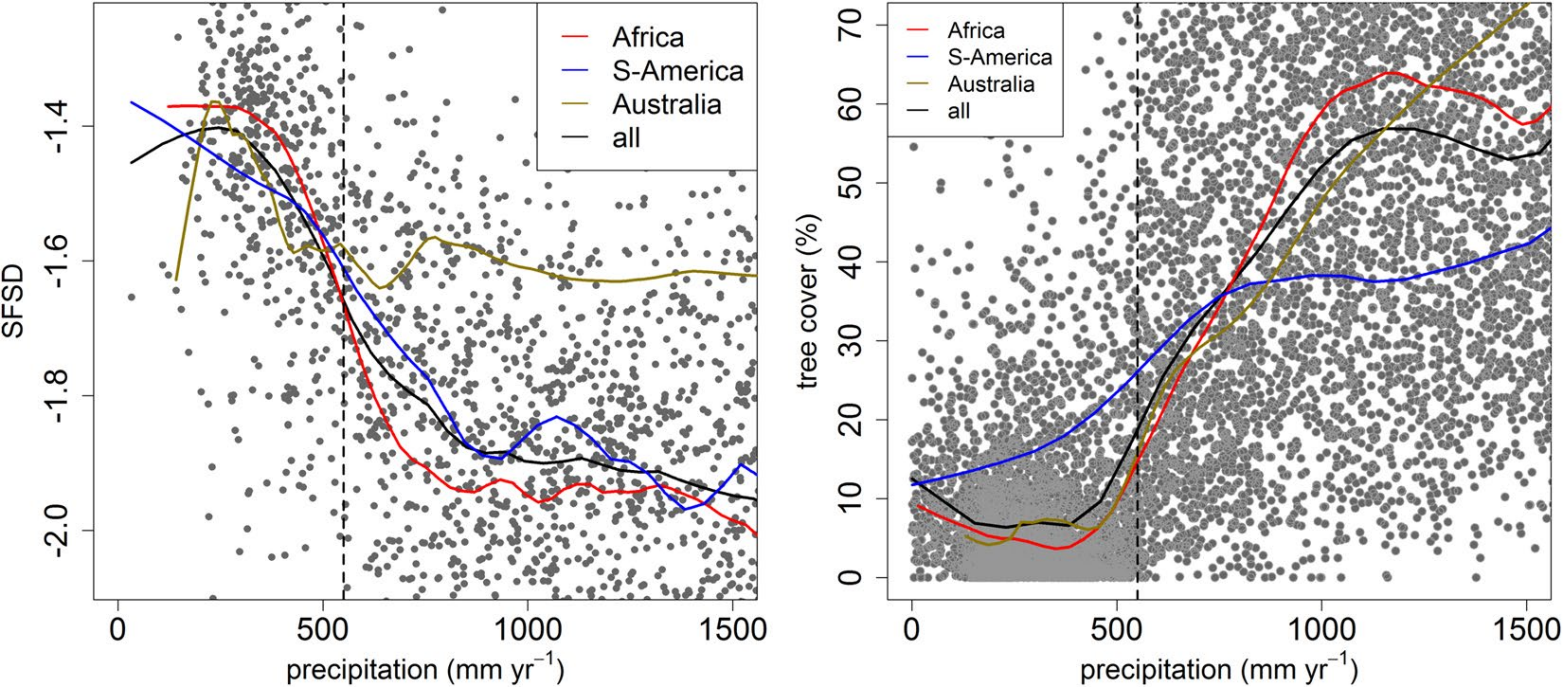
The slope of the fire size distribution (SFSD) over precipitation for the global tropics with the running means plotted as solid lines and the 550 mm precipitation threshold indicated (**a**). Tree cover is plotted over precipitation (**b**), with the coinciding shift at 550 mm indicated. Tree cover is derived from LiDAR data^19^ using the percentage of LiDAR footprints with vegetation height >5 m as an indicator of tree cover at 0.5° resolution (see supplementary material).

A closer look at the relationship between SFSD and rainfall reveals that the SFSD remains relatively constant until precipitation falls below ~550 mm where the SFSD rises to a markedly higher level (Figs 2a, S2). The same pattern can be observed when plotting SFSD over GPP (Gross Primary Production; see Fig. S3) with precipitation closely related the vegetation productivity over the tropics^17^. This shift in fire size distribution is clear and consistent for Africa, with South America following the same trend. The pattern is different for Australia, with large fires commonly found even at high precipitation levels (see also Supplementary Figs S4 and S5).

The precipitation level where the fire size distribution changes (~550 mm) corresponds roughly to the conditions where Hirota *et al*.^1^ (Fig. 1c therein) report the drier limit to the savanna (see also Sankaran *et al*.^6^). Scholes *et al*.^18^ also reported pronounced vegetation transitions for southern Africa around this precipitation threshold, with possible analogues in northern Africa, Australia and America. We analysed a new satellite LiDAR based estimate of tree cover^19^ around this climatic threshold (Supplementary methods). The LiDAR data confirm a drop in tree cover (Figs 2b, S2) and canopy height (Fig. S6) around 550 mm precipitation, indicating that this might be a critical precipitation threshold at which not only the fire regime, but also the vegetation changes sharply.

The difference in fire behaviour between the dry and wet tropics is not restricted to SFSD. In the drier tropics we also observe larger absolute mean and maximum fire sizes (95 percentile), and higher fire intensity in terms of Fire Radiative Power (FRP), whereas the number of fires and fire frequency (total burned area) are lower than in the wet tropics (Table 1, see Fig. S7 for figures with burned fraction and Fig. S8 for FRP). It should be noted that these values are relative to the tropical region since fire regimes with intenser fires are well known to occur in other biomes such as boreal forests. The distinctly different fire regime below 550 mm mean annual precipitation and the concomitant loss of trees around this threshold raise the question of how this intriguing change around a climatic threshold might be explained.

**Table 1:** The difference in fire characteristics between wet and dry tropics. The mean value for a set of fire indicators for the dry and wet tropics (550 mm precipitation threshold) is given. The difference in fire characteristics where tested with the Welch student t-test. SFSD = Slope of the Fire Size Distribution, FRP = Fire Radiative Power.

	SFSD	Mean fire size (ha)	Percentile95 fire size (ha)	Mean FRP (MW)	Number of fires (100 km^-2^)	Burned fraction (%)	Length of fire season (month)
Dry ( < 550 mm)	−1.501	929.668	3002.216	47.978	2.643	3.310	6.210
Wet ( > 550 mm)	−1.902	532.197	1466.396	31.424	17.478	8.839	7.149
t-value	31.391**	6.763**	11.498**	14.143**	−33.550**	−14.414**	−8.130**

**p < 1e-10.

The high frequency of fires in savannas may result from a combination of factors. Fire occurrence is highest at intermediate precipitation and thus vegetation productivity levels (the intermediate fire-productivity hypothesis^20^). In dry regions fires are limited by fuel availability while fires are largely absent from high precipitation regions because the fuel is generally to wet to burn^21^. At intermediate levels, fuels can build-up during the productive wet period. This fuel build-up is further exacerbated by the limited palatability of the grasses present in these systems^18^. During dry periods the high frequency of human-ignited fires leads to a peak in fire occurrence. The relative small size of such fires could be related to the heterogeneity of mesic landscapes, where the topographic complexity also increases around 550 mm annual precipitation (Fig. S9). When it comes to the possibility for fires to spread, moister places may act as barriers^22^ and thus keep savanna fires relatively small. When the climate is dry enough (around 550 mm, which would be a ‘percolation threshold’), these barriers would not prevent fire propagation anymore, allowing full Self Organized Criticality to develop. Fires can spread freely and are limited merely by the spatial pattern of fuel limitation resulting from earlier fires or grazing. In favour of this view is the fact that our observed SFSD for these dry areas are consistent with SOC fire models^23^, whereas the savanna fire regime we find is biased towards small fires.

The relative rarity of fires in dry places may also result from a combination of factors. Dry areas typically support low human population and lightning is less frequent, implying lower ignition rates^24^. However, more importantly, fuel accumulation is slower and strongly coupled to rainy pulses. Indeed, larger and intenser fire events over vast areas usually follow peaks in grass production during rainy phases associated with the El Niño Southern Oscillation^25^. It is possible that for some drylands around the world, a feedback between fire regime and shrub cover could also play a role in explaining the observed patterns. Below a certain fire frequency shrubs may invade grasslands^26–28^. Shrubs burn less readily than dry grass and further reduce fire occurrence. This may imply that once a fire occurs the accumulated fuel allows fire to burn more intensely and over large extents. Whereas savanna trees are well adapted to survive small and less intense fires^29^, more intense fires kill more trees and inhibit tree recruitment^12^, potentially explaining the conspicuously treeless state and amplifying the effect of decreasing precipitation. Overgrazing under dry conditions might strengthen this shift by facilitating shrub encroachment and shifts from perennial to annual grasses^27,30^. Furthermore, litter flammability properties tend to reinforce large scaled patterns in fire frequency and intensity^31^.

Many uncertainties remain when it comes to the complex interactions between vegetation, climate, fire, herbivores and humans^3,5,27–32^. Our observations may help to shape a search image for what happens at this transition to a treeless state. One observation that might guide further research is the fact that we observed a different pattern in Australia, where high SFSD values are found over the entire precipitation gradient. These more widespread large fires, even under high precipitation, seem to indicate different spatial fire behaviour, probably related to the previously noted low tree cover in Australia^1,2^. In addition, Australia differs from other continents by its limited orography, resulting in continuous and non-dissected landscapes even under mesic conditions, even further accentuated by the very low population density. This allows fire to spread over large areas contrary to mesic areas of other continents. This would be consistent with the hypothesis that the high SFSD found over savannas is due to the presence of widespread natural fire breaks, while SOC develops in areas where this is not the case.

The change to a RIB fire regime below 550 mm annual precipitation may have important consequences under future global change. Although the future projections of precipitation are still uncertain, there is a general trend of dry areas becoming drier under future climate change^33^. This implies that the 550 mm precipitation boundary might move into the classical savanna areas (Fig. S10), implying that fire regimes could change from low to high intensity for large areas such as southern Africa and the Gran Chaco region in South America, with concordantly a decrease in fire frequency. The impact will likely differ locally depending on spatial heterogeneity and differences in human land use practises^34^ with humans currently decreasing burnt area globally with unknown impacts on ecosystem dynamics^35^. Furthermore, other large scaled environmental changes might cause a disruption from the present fire regime. The high atmospheric CO_2_ levels cause an increase in vegetation productivity (CO_2_ fertilization) and tree cover with a variable response on fire occurrence^21,36^. These effects will also differ between continents. For instance, in Australia where RIB fires already dominate over the entire precipitation gradient, little change may be expected except for a reduction in fire frequency due to lower productivity^21^. By contrast, South America has much lower fire frequency than the other continents (Fig. S7). The factors that cause such differences are unknown^32^, but land use and other human environmental impacts may well play an important role. Although fire characteristics depend in part on human impacts^11^, the shift we find in most of the global tropics around 550 mm precipitation suggests that climate-induced change in fire characteristics can be profound. This may drive shifts from savannas to a treeless state, with resulting impacts on carbon stocks, biodiversity and ecological services. At the same time the change into a RIB fire dominated state would imply an altered fire risk to society in some regions.

## Methods

The fire regime is described by the frequency, intensity and size of fires. Fire frequency is indicated by the % of the gridcell burned as detected by satellite derived burned-area from the GFED4^37^. As a proxy for fire intensity we use satellite derived fire radiative power from the MODIS thermal anomaly product^38^. The fire size was estimated by the Slope of the Fire Size Distribution (SFSD) based on the global MODIS MCD45 burned area data^16^ adapted to estimate individual fire sizes^39,40^ (see Supplementary Information). Next to the SFSD, we also extracted the mean fire size, 95 percentile fire size from the same dataset as additional information on fire size behaviour. Further fire regime indicators used are the annual fire number for each grid cell^40^. The length of the fire season (number of months with >10% of annual burned area) was extracted from the MODIS thermal anomaly product^38^. These datasets were aggregated at 1° × 1° spatial resolution for analysis.

When plotting the SFSD over precipitation, a clear shift at 550 mm mean annual precipitation^41^ was detected for the total dataset and for each continent separately. We detected the precipitation threshold of these transitions using a simple and robust method using the difference in mean SFSD (or tree cover) above and below a given precipitation level (with equal sample size both above and below the precipitation value selected; 200 and 1000 samples each side, for SFSD and tree cover respectively). If a transition exists this should show up as a peak difference in mean SFSD (or tree cover) over the precipitation gradient, with the peak being the precipitation value at which the shift occurs (Fig. S2). The range of high values/transition overlaps strongly, with the peak around 550 mm which is used as the threshold value.

We tested whether the SFSD and a set of other indicators of fire characteristics (mean fire size, 95 percentile fire size, fire radiative power, number of fires and burned fraction) extracted from existing global fire products showed a shift at this threshold using the Welch student t-test. This threshold at 550 mm precipitation seems to coincide with a shift from treeless vegetation to savanna. This was analysed using the satellite based LiDAR vegetation height measurements^19^. See supplementary methods for more information.

### Data availability

All the datasets generated during and/or analysed during the current study are available from the corresponding author on reasonable request.

## Electronic supplementary material

supplementary information
