# Supplementary material for: Rare, Intense, Big fires dominate the global tropics under drier conditions
Source: Sci Rep. 2017 Oct 30;7:14374. doi: 10.1038/s41598-017-14654-9 (PMC5662699; doi:10.1038/s41598-017-14654-9)

### Supplementary information

### Rare, Intense, Big fires dominate the global tropics under drier conditions

Stijn Hantson^1*^, Marten Scheffer^2^, Salvador Pueyo^3^, Chi Xu^4^, Gitta Lasslop^5^, Egbert H. van Nes^2^, Milena Holmgren^6^, John Mendelsohn^7^

### ^1^ Karlsruhe Institute of Technology, Institute of Meteorology and Climate research, Atmospheric Environmental Research, 82467 Garmisch-Partenkirchen, Germany.

^2^ Environmental Sciences Department, Wageningen University, Wageningen, The Netherlands.

### ^3^ Dept. de Biologia Evolutiva, Ecologia i Medi Ambient, Universitat de Barcelona, Av. Diagonal 645, 08028 Barcelona, Catalonia, Spain.

### ^4^ School of Life Sciences, Nanjing University, 210093 Nanjing, China.

^5^ Max Planck Institute for Meteorology, Fire in the Earth System, 20146 Hamburg, Germany.

^6^ Resource Ecology Group, Wageningen University, Wageningen, The Netherlands.

^7^ Research and Information Services of Namibia (RAISON), PO Box 1405, Windhoek, Namibia

* Correspondence to stijn.hantson@kit.edu

**Supplementary methods**

**1 Data processing and sources**

A number of datasets of fire characteristics and environmental factors were assembled for the tropical region (17N-35S) at 1x1° spatial resolution. All areas with more than 25% agricultural land cover ^1^ or for which the fire size distribution could not be calculated were excluded from analysis.

*Slope of the fire size distribution (SFSD)*

We used the MCD45 MODIS global burned area product for the period 2002-2010. Fire patches are separated in individual fires by combining adjacent burned gridcells within a certain time window to one fire following ^2^ but with a 14 day instead of 8 day time step ^3^. The resulting dataset is a global dataset with the fire size for each individual fire detected. A linear regression was fitted to the log-transformed fire size frequency data of each 1x1° grid cell. The bins of fire size have the same width at a logarithmic scale, with the number of fires normalized over the bin size following ^4^ and number of fires calculating frequency densities
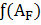
 as:


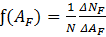
 (S1)

Where
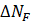
 are the number of fires within a bin with width
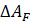
. When less than 3 fires were present in a certain bin, this bin was joined to the previous bin. The -
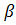
 value of the power law (Eq. 1) is used to indicate the SFSD, so that an increase in SFSD indicates an increase in frequency of large fires. Two examples for Australia with high SFSD and Angola with low SFSD are presented in figure S1.

*Other datasets used*

Tree cover: The tree cover was extracted from the MODIS vegetation continuous fields, MOD44 collection 3 product ^4^ for the year 2001. Hirota et al. ^5^ determined the threshold between treeless and savanna state at 5% tree cover, which was followed in this study. Due to the possible different function of wet and dry tropical ecosystems we separated dry and wet areas based on a 550 mm rainfall threshold detected in the present study (see previous paragraph and Fig S2). An independent estimate of tree cover was extracted from LiDAR satellite data (see Tree height) for figure 2b.

Tree height: Tree height data was extracted from a global vegetation height product at 0.5° resolution ^7^. This remote sensing product estimated vegetation height distribution from 0 to 70 m in 0.5 m intervals per 0.5° × 0.5° grid cell using LiDAR measurements collected by the Geoscience Laser Altimeter System (GLAS) on the Ice, Cloud and land Elevation Satellite (ICESat) during 2003–2009. We calculated the mean vegetation height within each cell. The tree cover fraction was calculated as the fraction of LiDAR footprints with vegetation height >5m. We filtered out dense forests with tree cover > 60% since we want to focus on the treeless-savanna transition. Data from areas under human landuse and water were also excluded from analysis based on the Global Land Cover 2000 dataset ^8^.

Precipitation: We used Precipitation data as distributed by the Global Precipitation and Climatology Centre (GPCC) at 0.5deg ^9^ for all analysis, except figure 1 where we used the 1 km^2^ precipitation from the worldclim dataset ^10^. This downscaled data is available for current conditions (1950-2000) and future projections. As an example of future projections we used the CMIP-5 HadGEM2-ES precipitation outcome for 2070 (2061-2080) based on the RCP6 Representative Concentration Pathway.

Number of fires: The number of individual fires extracted from the MCD45 MODIS product for the period 2002-2010 as explained above.

Mean and 95 percentile fire size: Using individual fires from the MCD45 product as explained above, the mean and the 95 percentile fire size are calculated for each grid cell in hectares.

Percentage burned area: The GFED4 MODIS based burned area estimates at 0.5° for 2002-2010 is used ^11^.

Mean FRP: The mean Fire Radiative Power indicates the energy released by the wildland fires as detected by MODIS active fire product in MW ^12^ .

Length of the fire season: The number of months with significant fire activity (>10% of total annual fire counts) as observed by TERRA-MODIS active fire product ^12^.

Topographic ruggedness index: The topographic ruggedness was calculated from a digital elevation model at 15” resolution (http://www.viewfinderpanoramas.org/dem3.html), calculating the difference in altitude between each cell and its surrounding cells using the gdaldem function the R package gdalUtils ^13^. The topographic ruggedness for each gridcell was estimated by taking the log10 of the mean ruggedness+1 within each gridcell.

Gross Primary Production (GPP) was extracted from the upscaling FLUXNET measurements ^14,15^, taking the mean of the six available datasets each creased using a different method.

**References**

1 Ramankutty, N., Evan, A. T., Monfreda, C. & Foley, J. A. Farming the planet: 1. Geographic distribution of global agricultural lands in the year 2000. *Glob. Biogeochem. Cycle* **22**, GB1003 (2008).

2 Archibald, S. & Roy, D. P. Identifying Individual Fires from Satellite-Derived Burned Area Data. Int. Geosci. Remote Se. **1-5**, 1462-1465 (2009).

3 Hantson, S., Pueyo, S., and Chuvieco, E.: Global fire size distribution is driven by human impact and climate, Global Ecology and Biogeography, 24, 77-86, 10.1111/geb.12246, 2015.

3 Grassberger, P. & Manna, S. S. Some More Sandpiles. *J Phys-Paris* **51**, 1077-1098 (1990).

4 Hansen, M. *et al.* Global percent tree cover at a spatial resolution of 500 meters: First results of the MODIS vegetation continuous fields algorithm. *Earth Interact.* **7**, 1-15 (2003).

5 Hirota, M., Holmgren, M., Van Nes, E. H. & Scheffer, M. Global Resilience of Tropical Forest and Savanna to Critical Transitions. *Science* **334**, 232-235 (2011).

6 Holmgren, M., Hirota, M., van Nes, E. H. & Scheffer, M. Effects of interannual climate variability on tropical tree cover. *Nat. Clim. Change* **3**, 755-758 (2013).

7 Los, S. O. *et al.* Vegetation height and cover fraction between 60° S and 60° N from ICESat GLAS data. *Geosci. Model Dev.* **5**, 413-432 (2012).

8 Bartholomé, E. & Belward, A. GLC2000: a new approach to global land cover mapping from Earth observation data. *Int. J. Remote Sens.* **26**, 1959-1977 (2005).

9 Schneider, U. *et al.* GPCC full data reanalysis version 6.0 at 0.5: monthly land-surface precipitation from rain-gauges built on GTS-based and historic data. doi: 10.5676/DWD_GPCC. FD_M_V6_050 (2011).

10 Hijmans, R. J., Cameron, S. E., Parra, J. L., Jones, P. G. & Jarvis, A. Very high resolution interpolated climate surfaces for global land areas. *Int. J. Climatol.* **25**, 1965-1978 (2005).

11 Giglio, L., Randerson, J. T. & Werf, G. R. Analysis of daily, monthly, and annual burned area using the fourth generation global fire emissions database (GFED4). J. Geophys. Res.- Biogeo. 118, 2169-8961 (2013).

12 Giglio, L. MODIS Collection 5 Active Fire Product User's Guide, version 2.4, http://modis-fire.umd.edu/Documents/MODIS_Fire_Users_Guide_2.4.pdf. (2010).

13 Greenberg, J. and Mattiuzzi, M. (2015). gdalUtils: wrappers for the Geospatial Data Abstraction Library (GDAL) Utilities. R package version 2.0.1.7. http://CRAN.R-project.org/package=gdalUtils

14 Jung, M., Reichstein, M., Schwalm, C. R., Huntingford, C., Sitch, S., Ahlström, A., Arneth, A., Camps-Valls, G., Ciais, P., Friedlingstein, P., Gans, F., Ichii, K., Jain, A. K., Kato, E., Papale, D., Poulter, B., Raduly, B., Rödenbeck, C., Tramontana, G., Viovy, N., Wang, Y.-P., Weber, U., Zaehle, S., and Zeng, N.: Compensatory water effects link yearly global land co2 sink changes to temperature, Nature, 541, 516-520, 2017.

15 Tramontana, G., Jung, M., Schwalm, C. R., Ichii, K., Camps-Valls, G., Ráduly, B., Reichstein, M., Arain, M. A., Cescatti, A., Kiely, G., Merbold, L., Serrano-Ortiz, P., Sickert, S., Wolf, S., and Papale, D.: Predicting carbon dioxide and energy fluxes across global fluxnet sites with regression algorithms, Biogeosciences, 13, 4291-4313, 10.5194/bg-13-4291-2016, 2016.

**Supplementary figures**

Figure S1: Two examples of the estimation of the fire size distribution for an area in Australia (a: high SFSD) and Angola (b: low SFSD). Here we plot the fire size frequency distribution which follows a power law, linearized in the log-log space.

Figure S2: Shift detection in tree cover and SFSD over mean annual precipitation. Differences in mean SFSD and tree cover below and above a certain precipitation value, scaled so that the maximum values are 1. Maximum value for SFSD is at 525 mm and for tree cover at 560 mm.

Figure S3: SFSD over gross primary production (GPP) plotted.

Figure S4: SFSD over precipitation for the different continents and a combination of all three with the moving mean plotted.

Figure S4b: SFSD over log 10 precipitation for the different continents and a combination of all three with the moving mean plotted.

Figure S5: SFSD over tree cover for the different continents and a combination of all three with the moving mean plotted.

Figure S6: Mean vegetation height over mean annual precipitation indicating a change in vegetation structure around 550 mm.

Figure S7: Mean annual burned area (%) over precipitation, separated for each continent and an overview with all data points including lines for the 90 percentile for each continent and all data.

Figure S8: Mean fire radiatiave power (FRP) from the MODIS thermal anomaly product in MW over precipitation for the tropics.

Figure S9: Mean topographic ruggedness within each gridcell over mean annual precipitation.

Figure S10: The possible distribution of treeless areas based on a 550 mm precipitation threshold for current conditions (a) and the difference with the future projection for 2070 by HadGEM2-ES under the RCP6.0 scenario as an example (b). In red areas which are currently >550 mm but are under this scenario projected to be <550 mm precipitation. This figure was produced using ArcMAP 10.2 http://desktop.arcgis.com/de/arcmap/ .

Figure S1


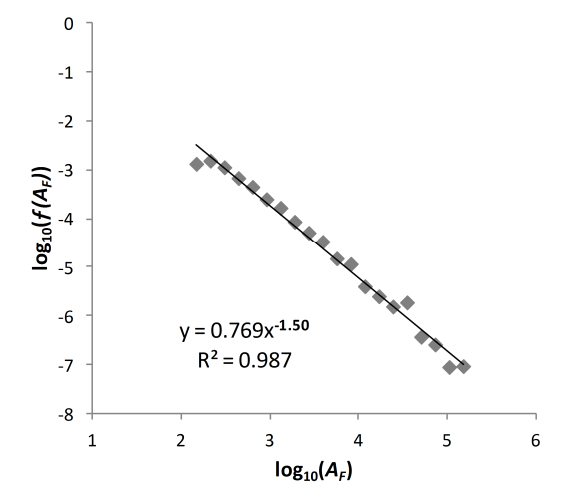

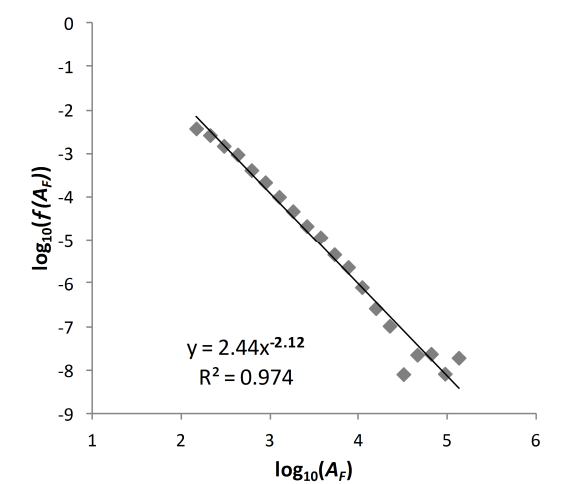


Figure S2


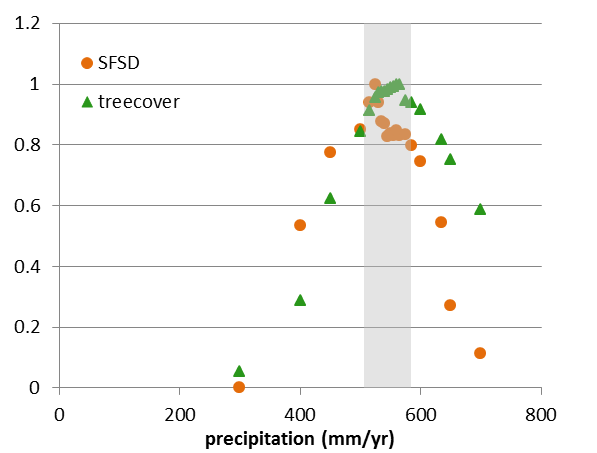


Figure S3


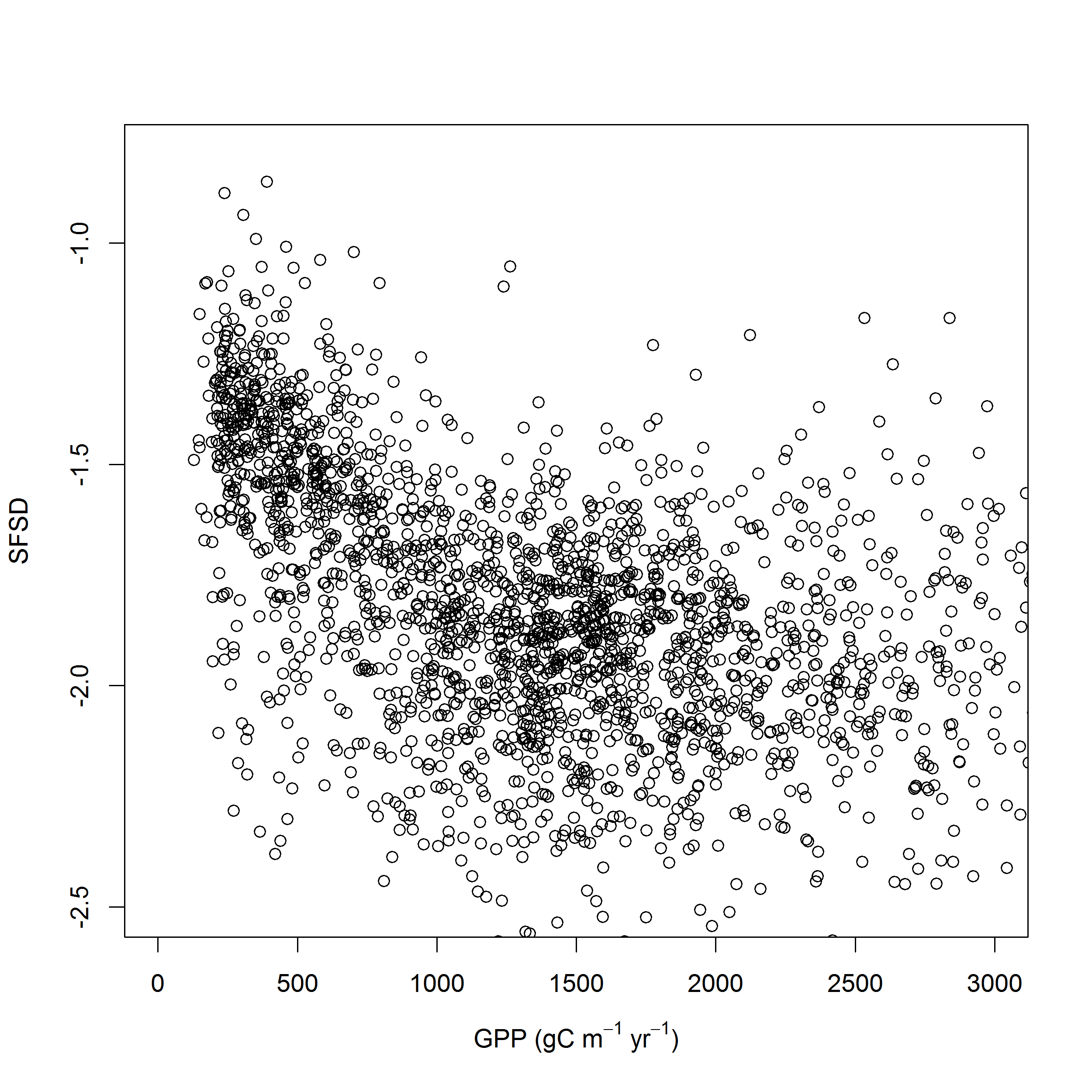


Figure S4


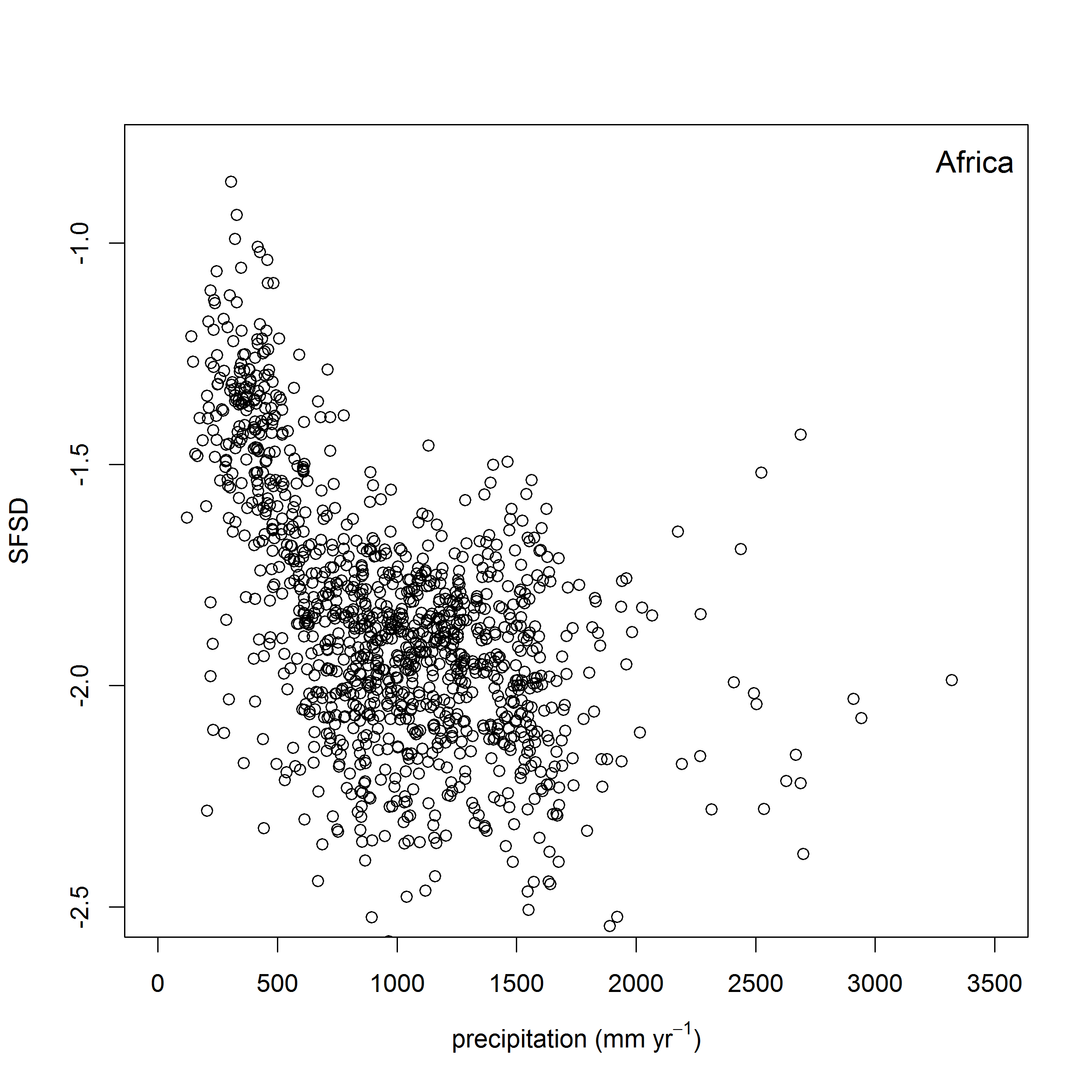

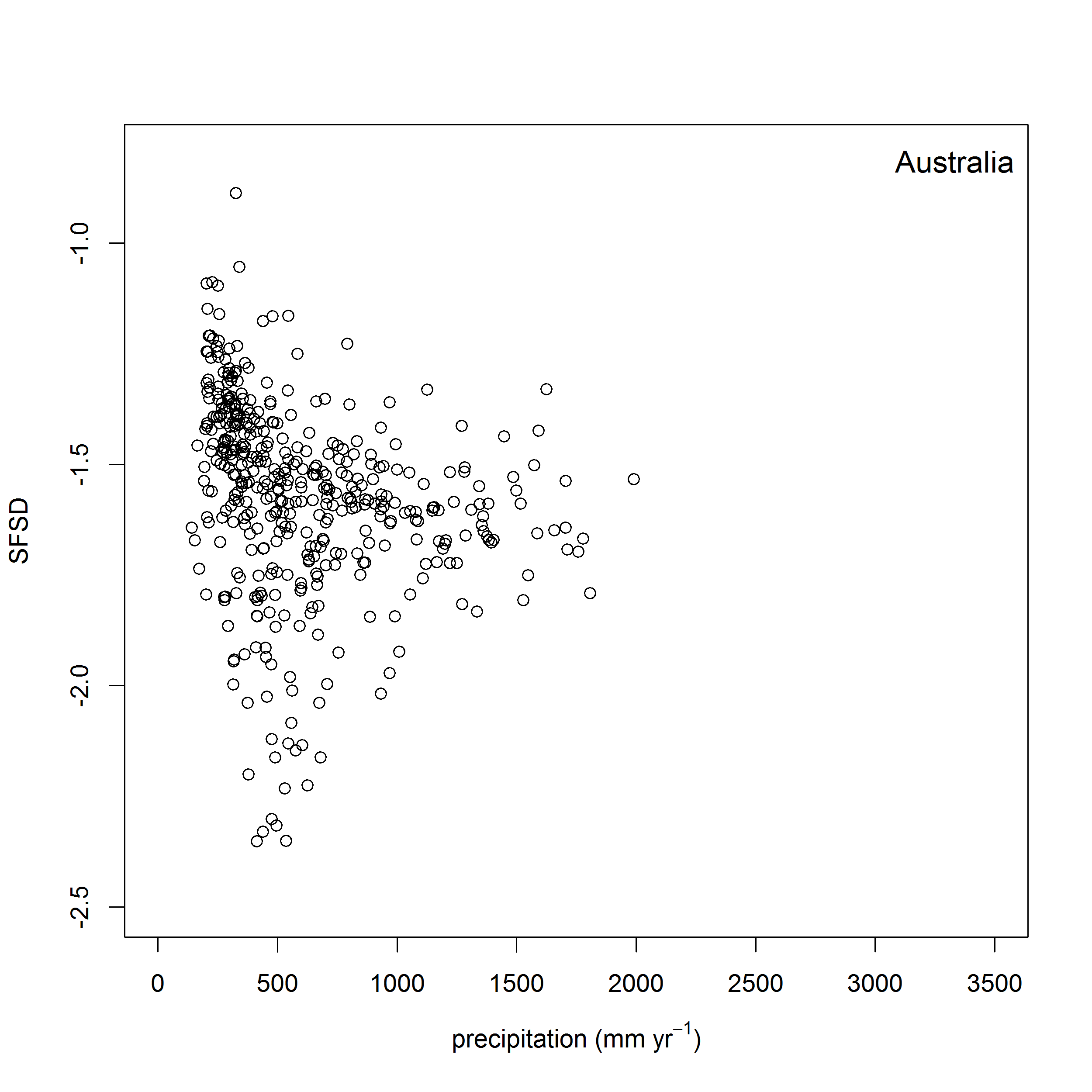

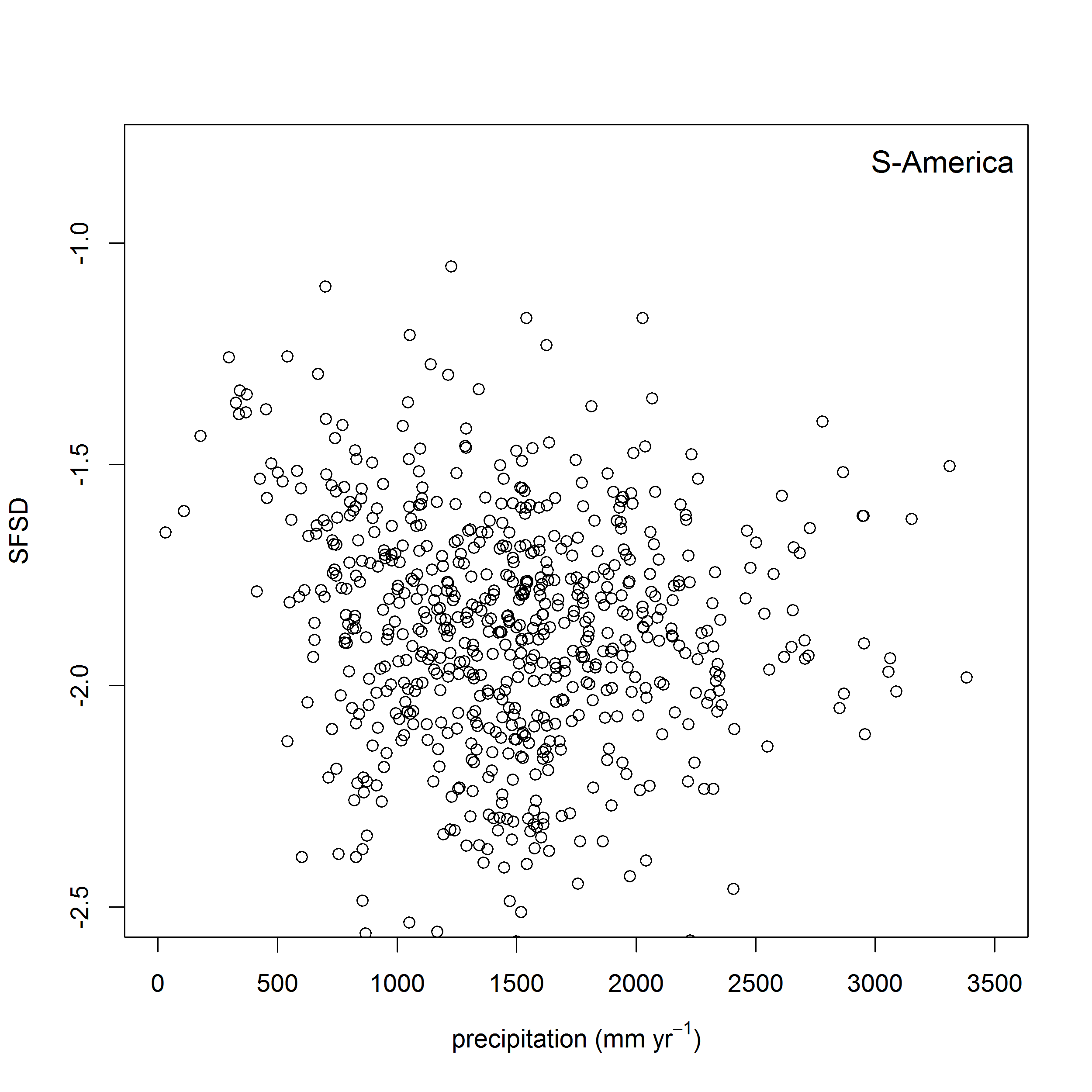

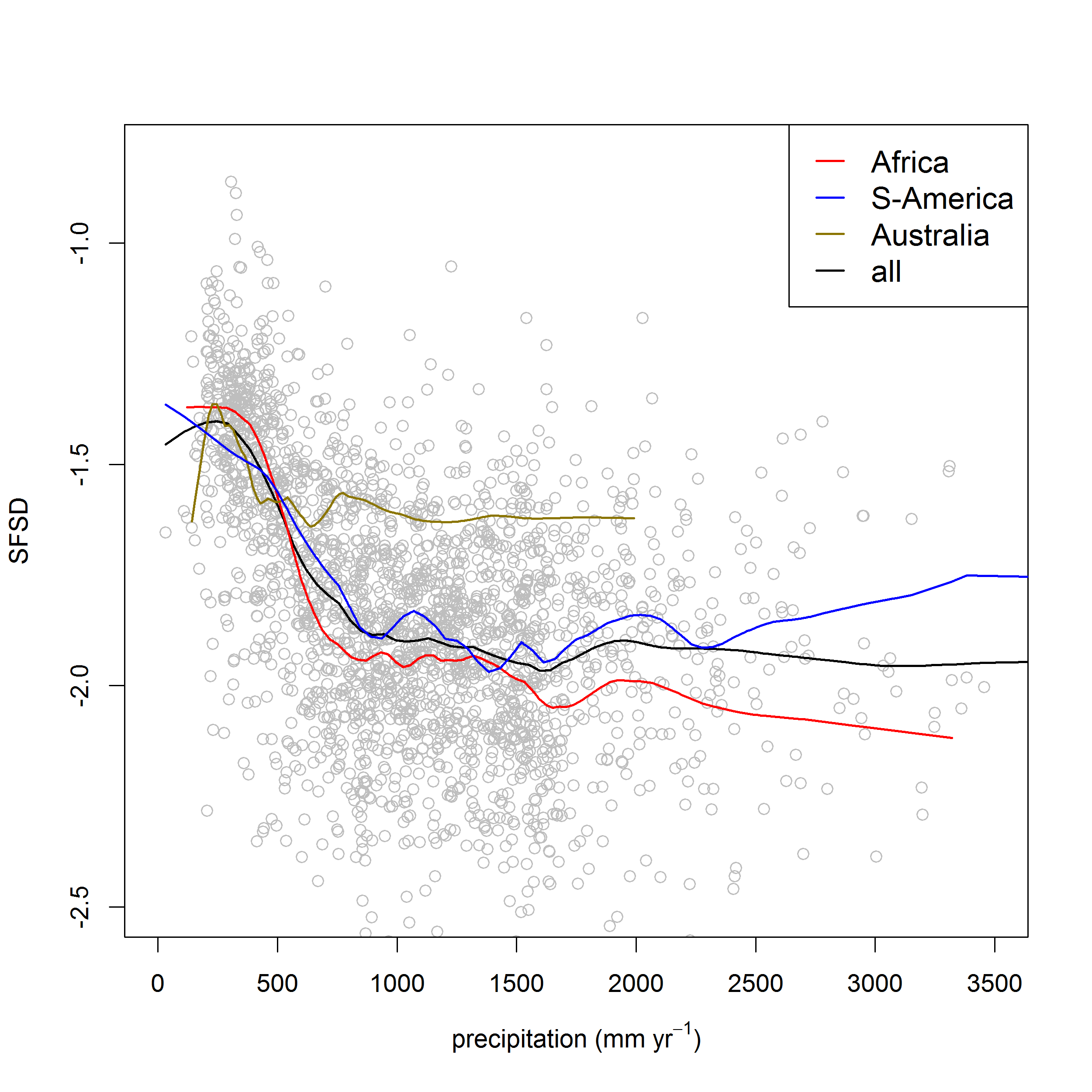


Figure S4b


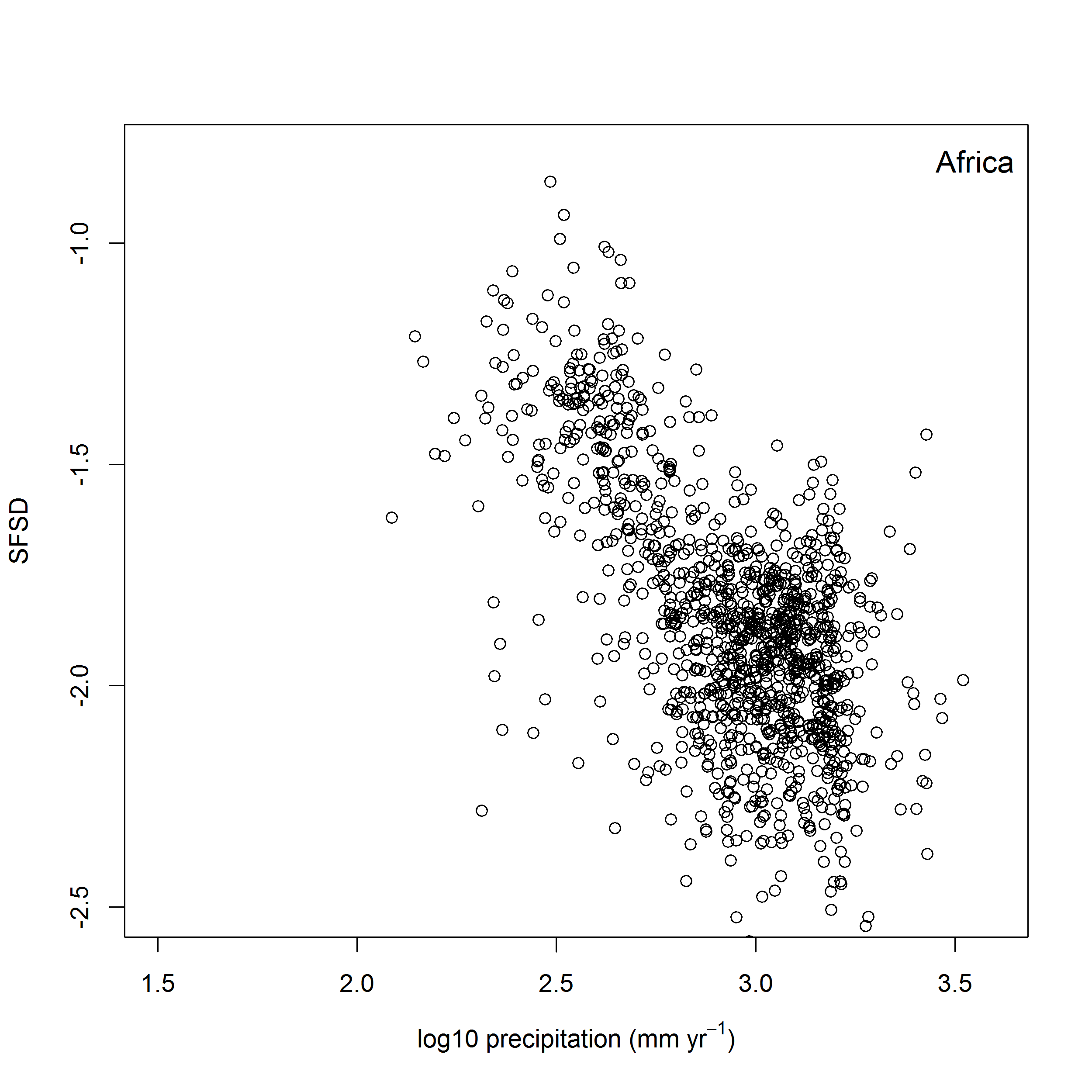

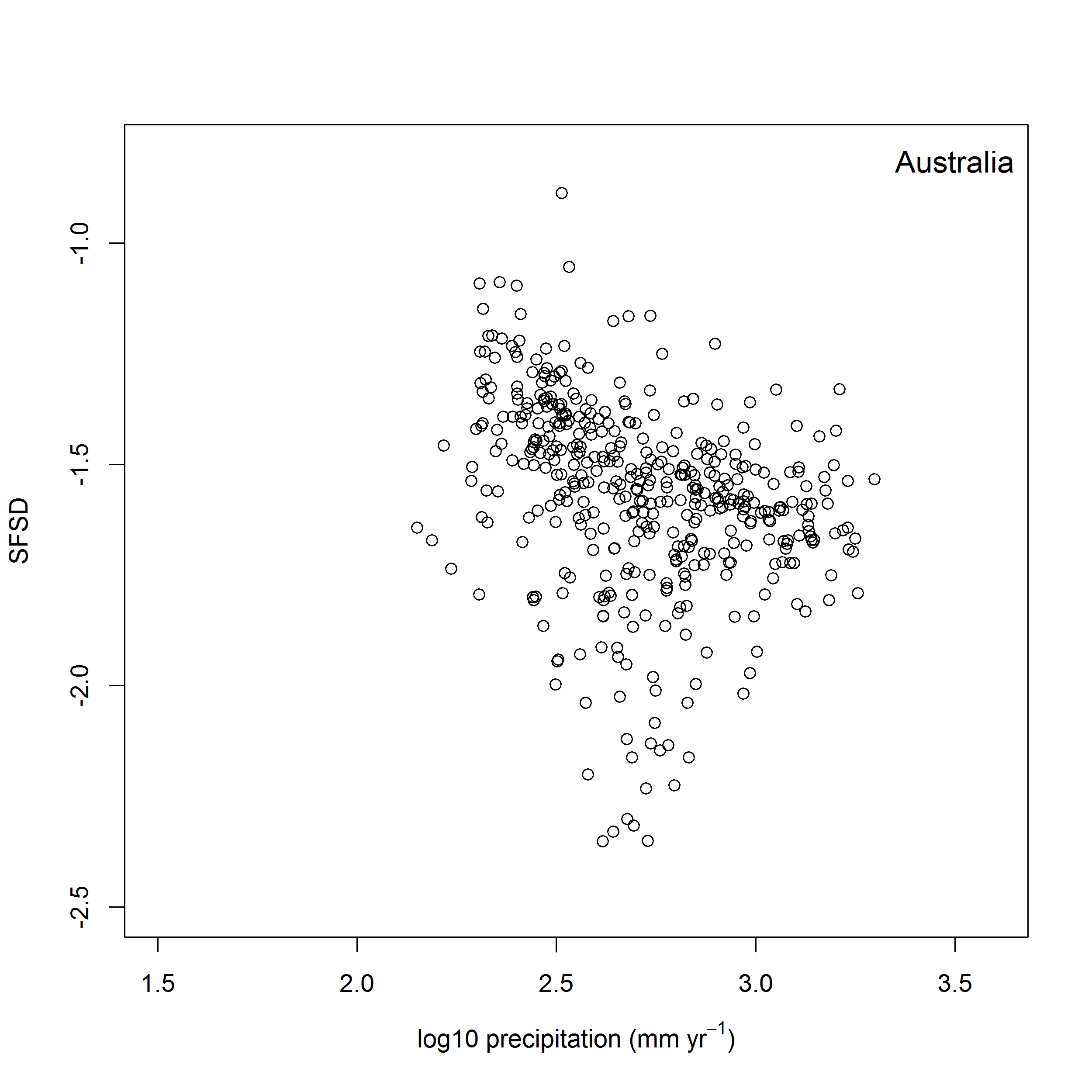

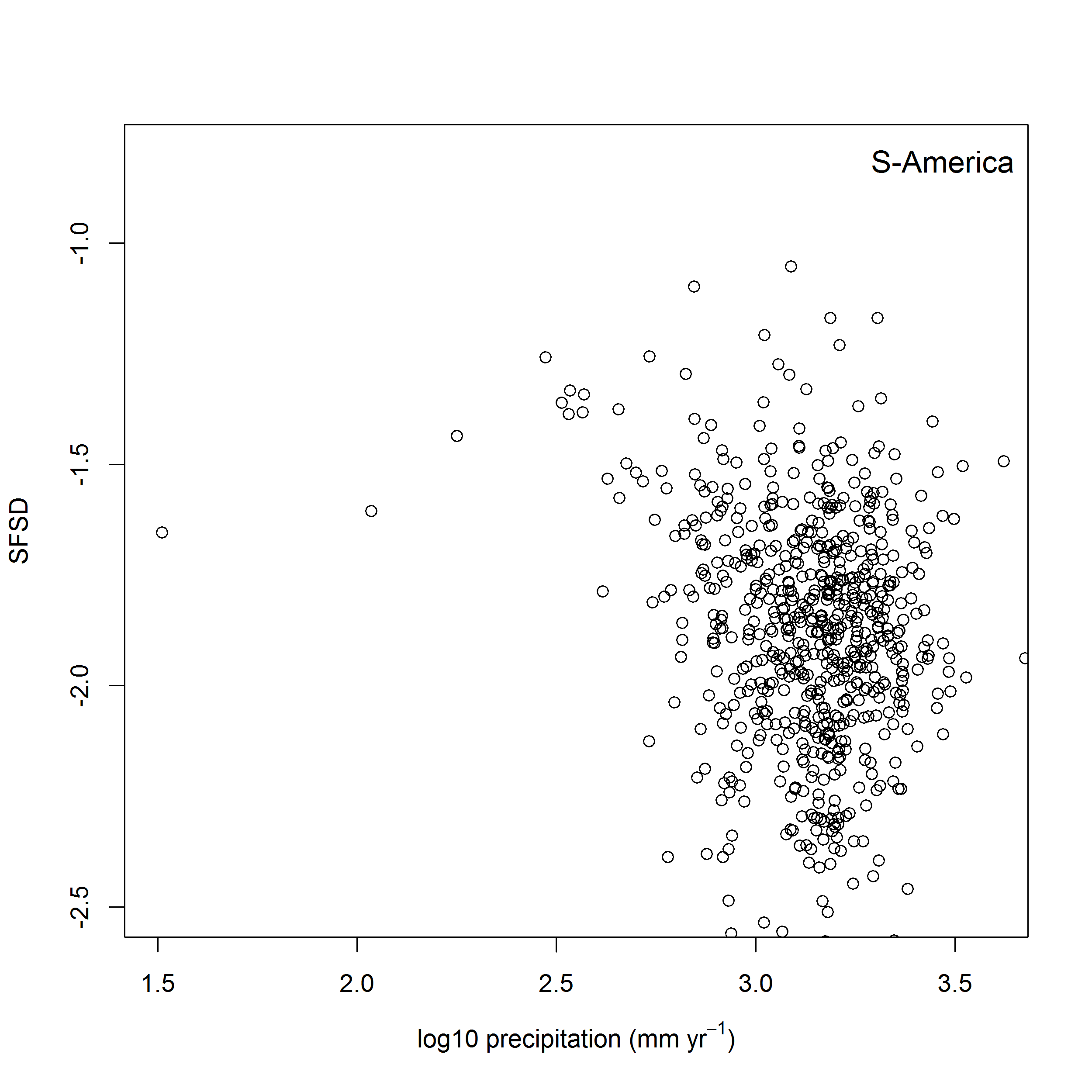

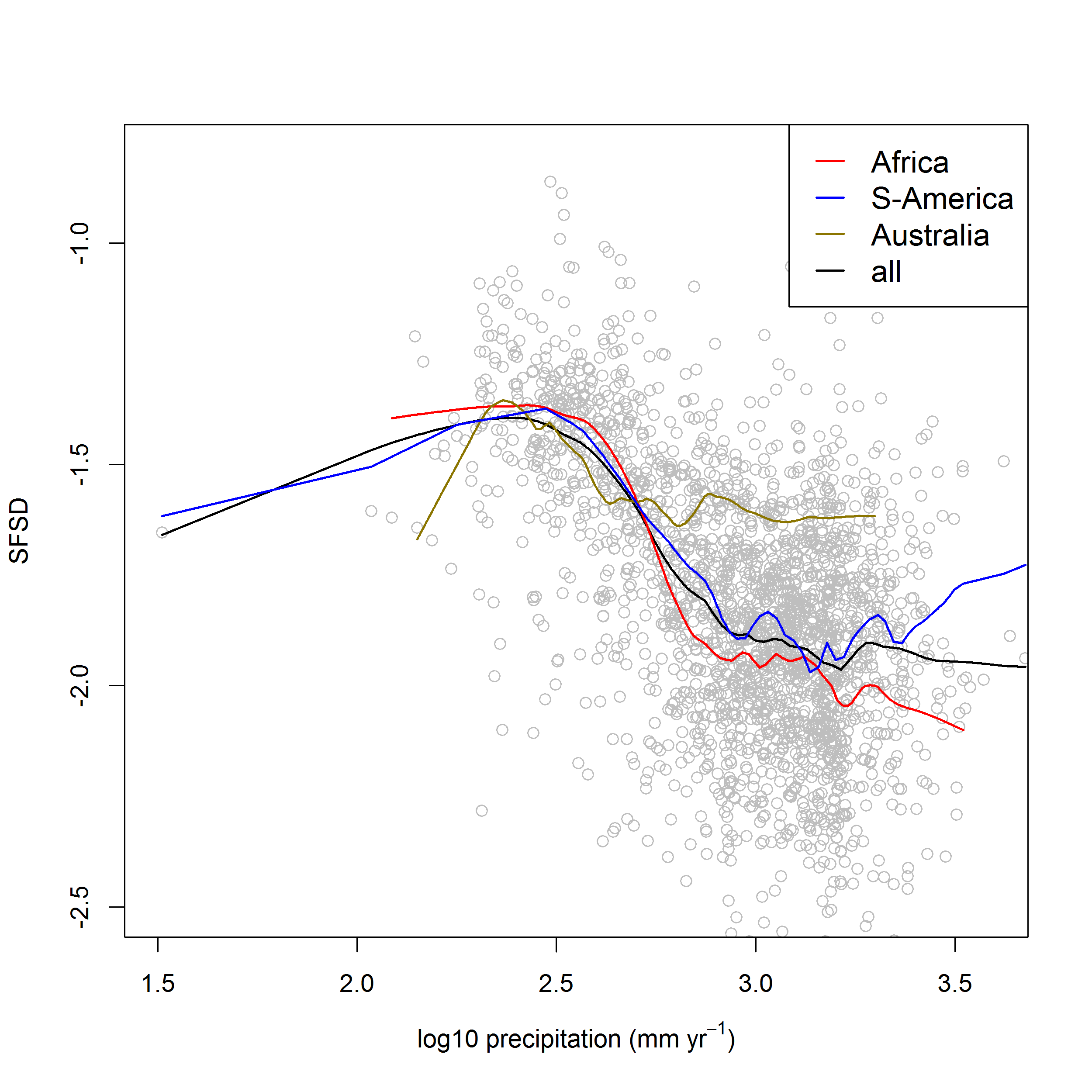


Figure S5


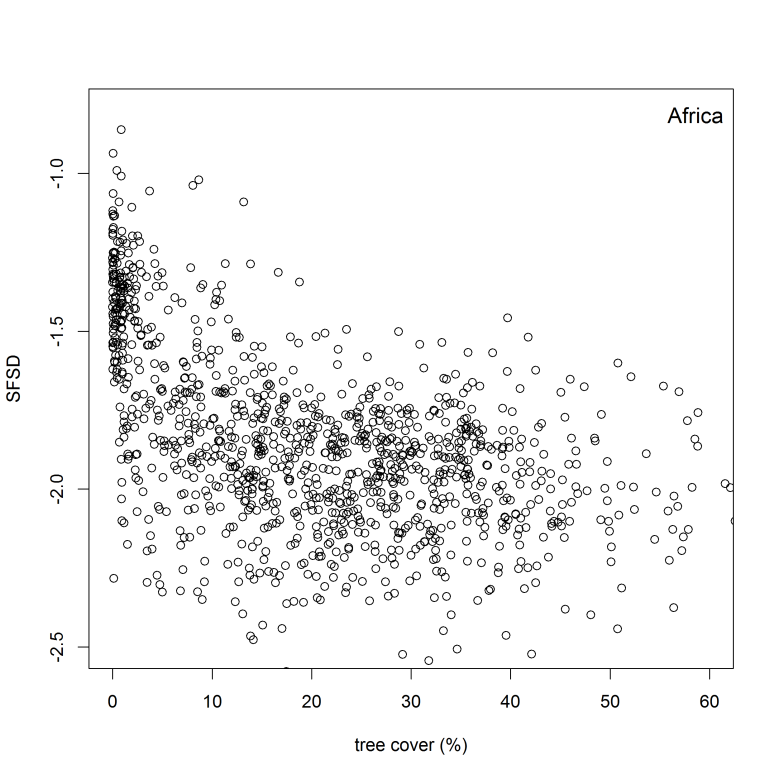

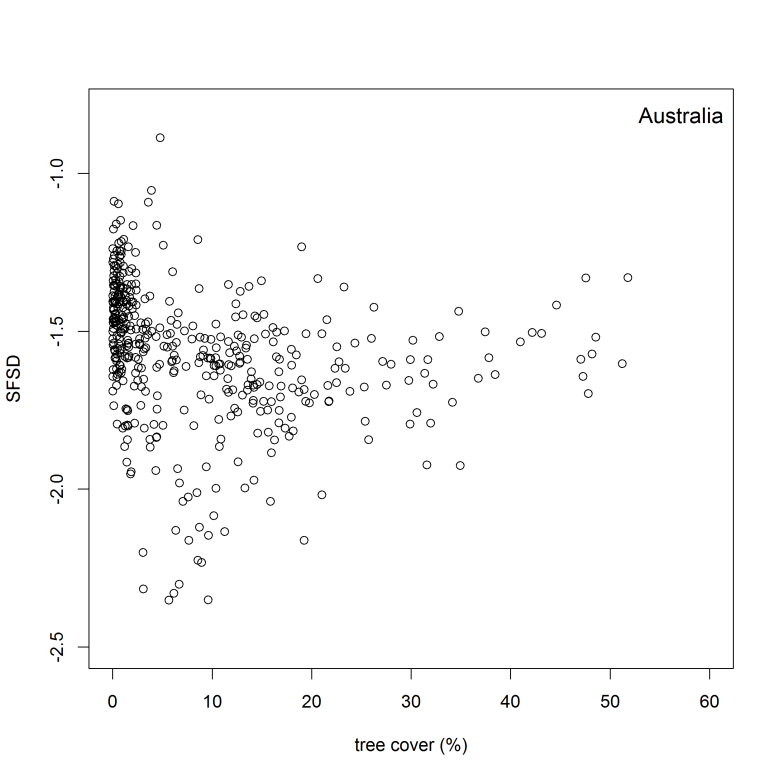

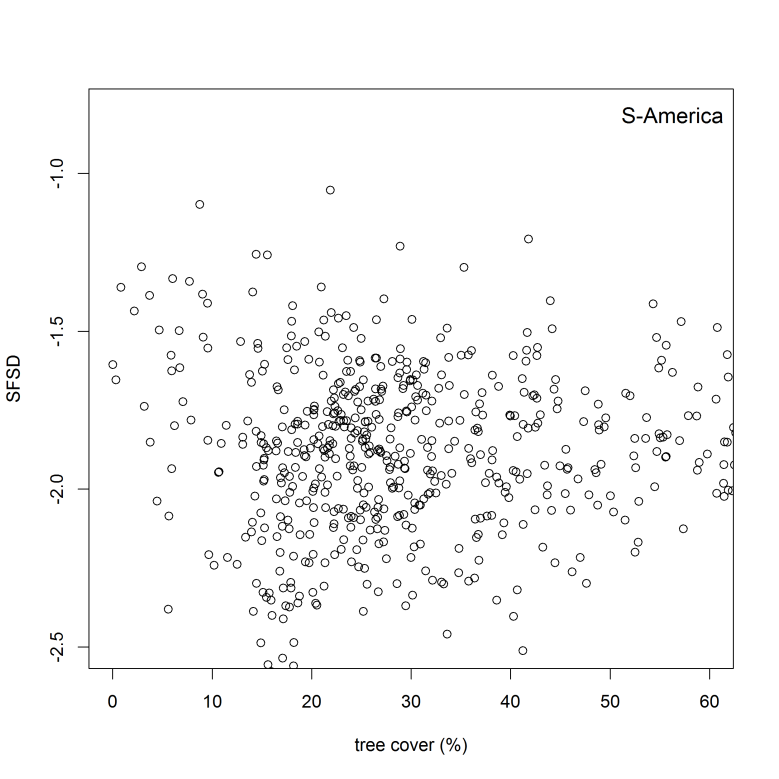

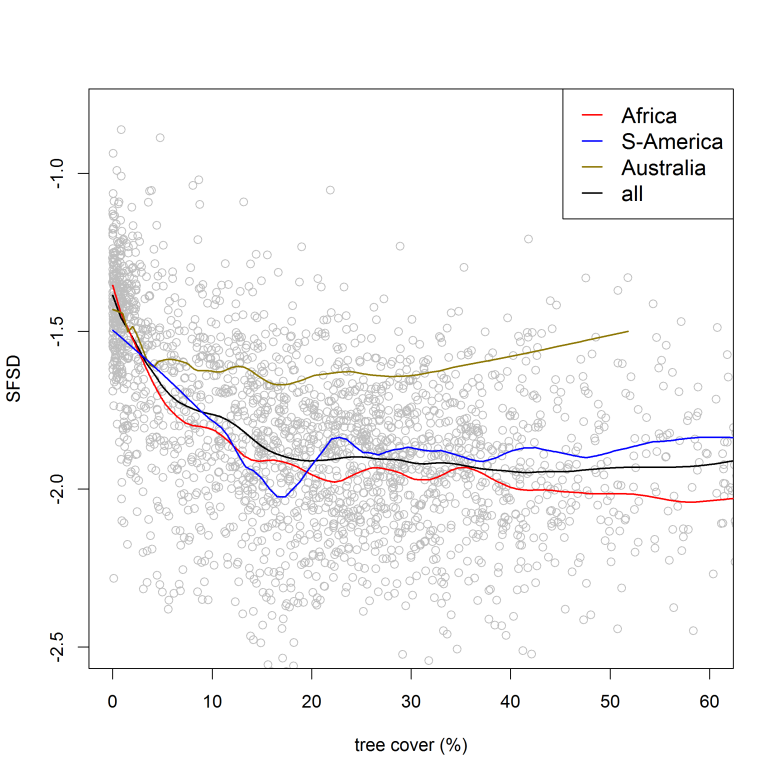


Figure S6


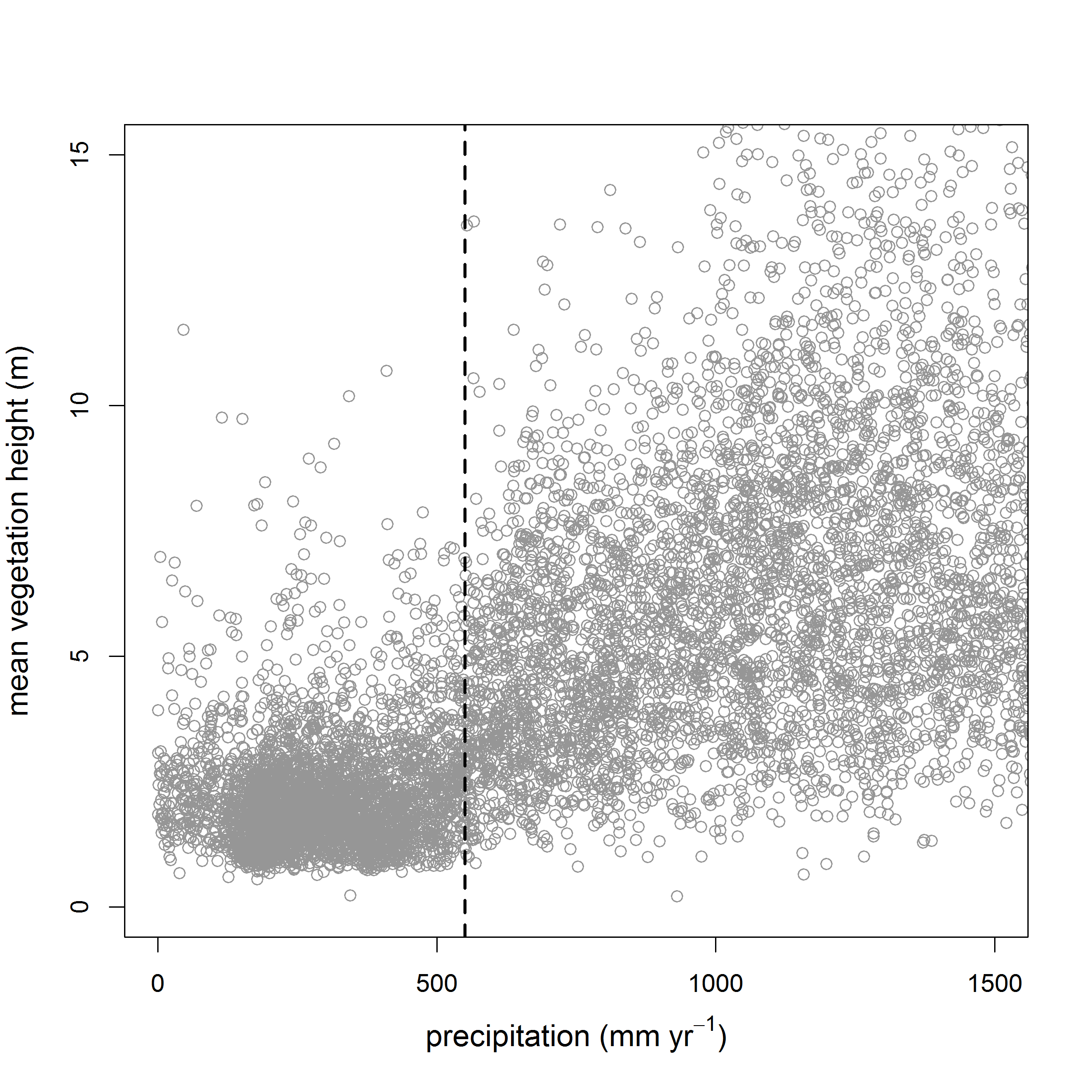


Figure S7


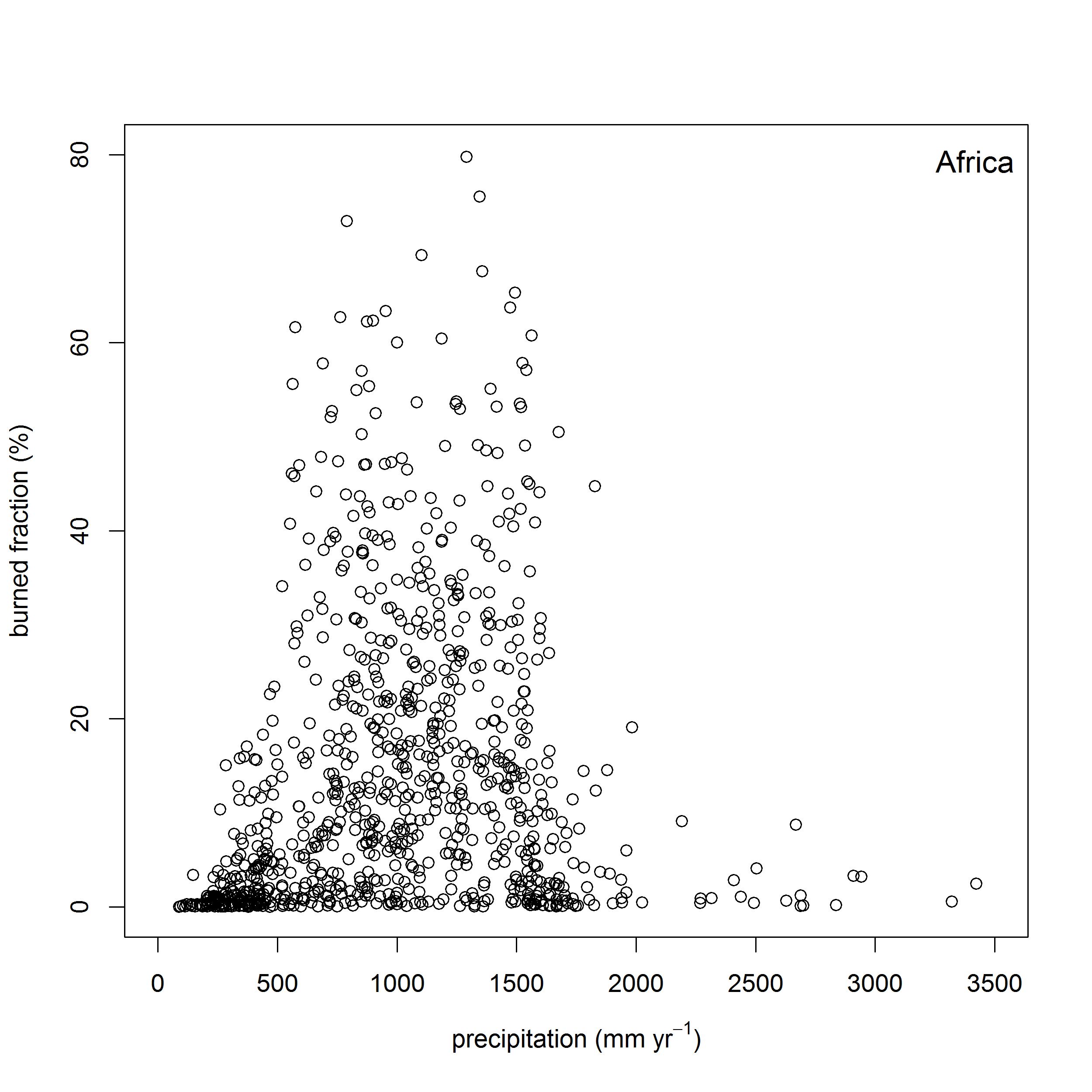

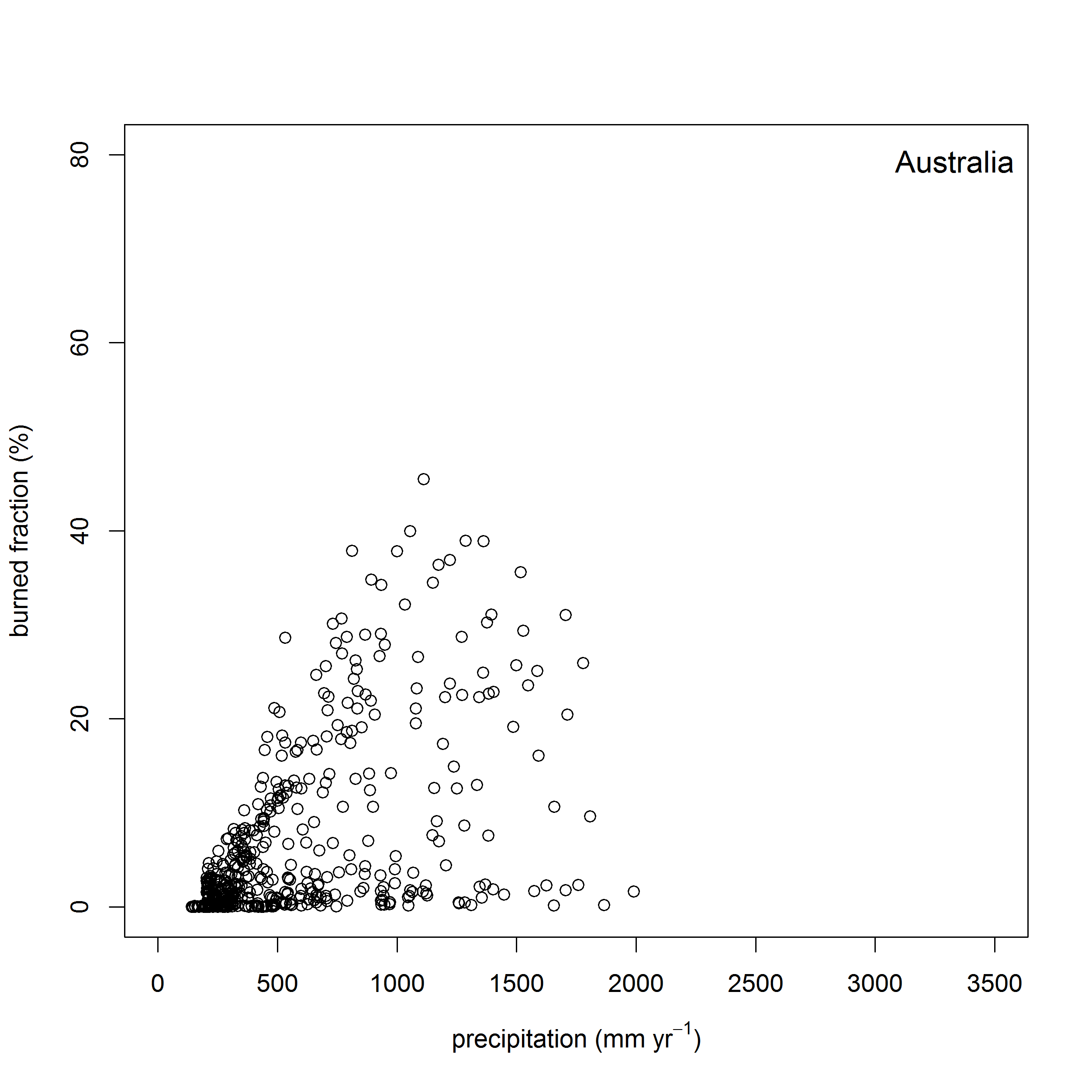

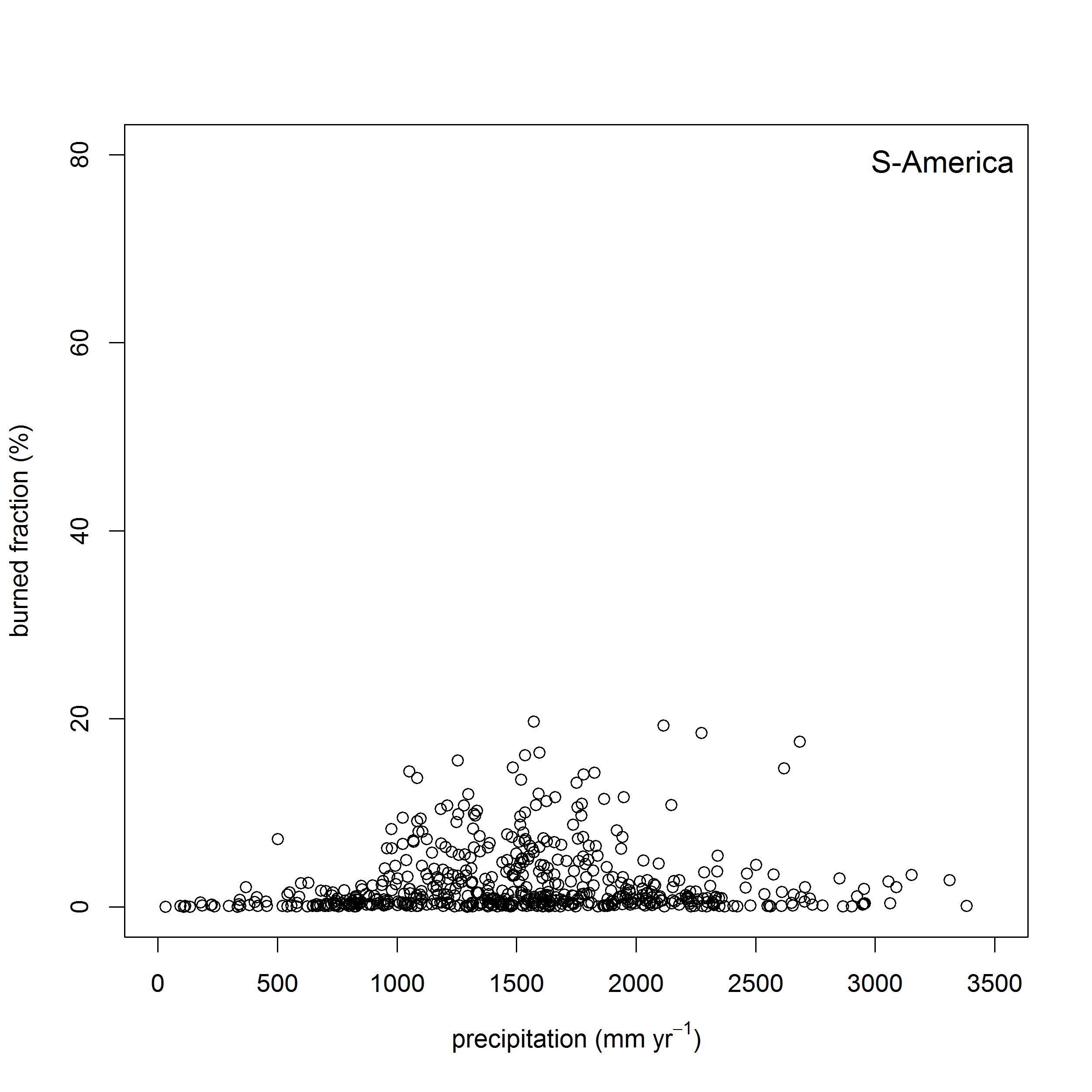

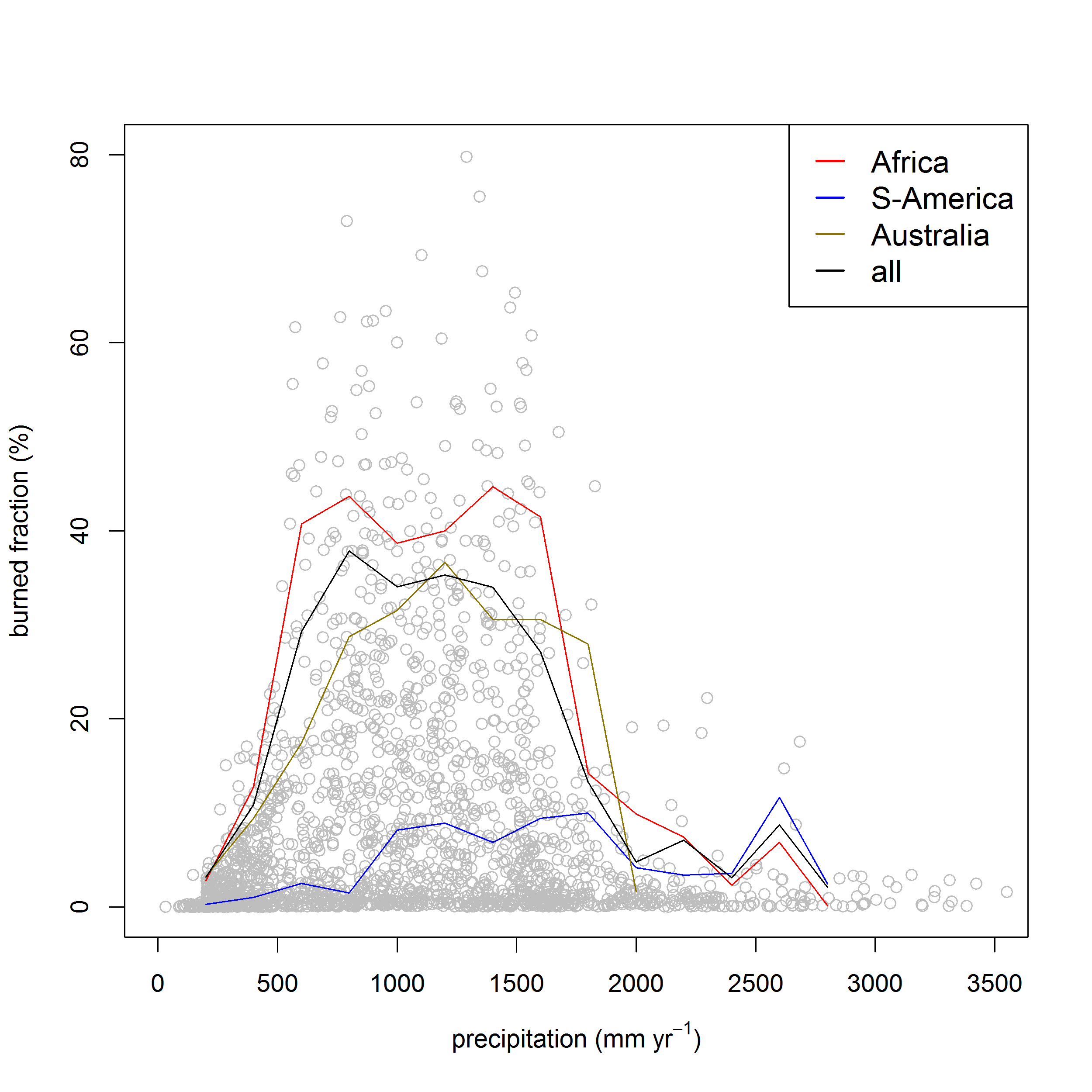


Figure S8


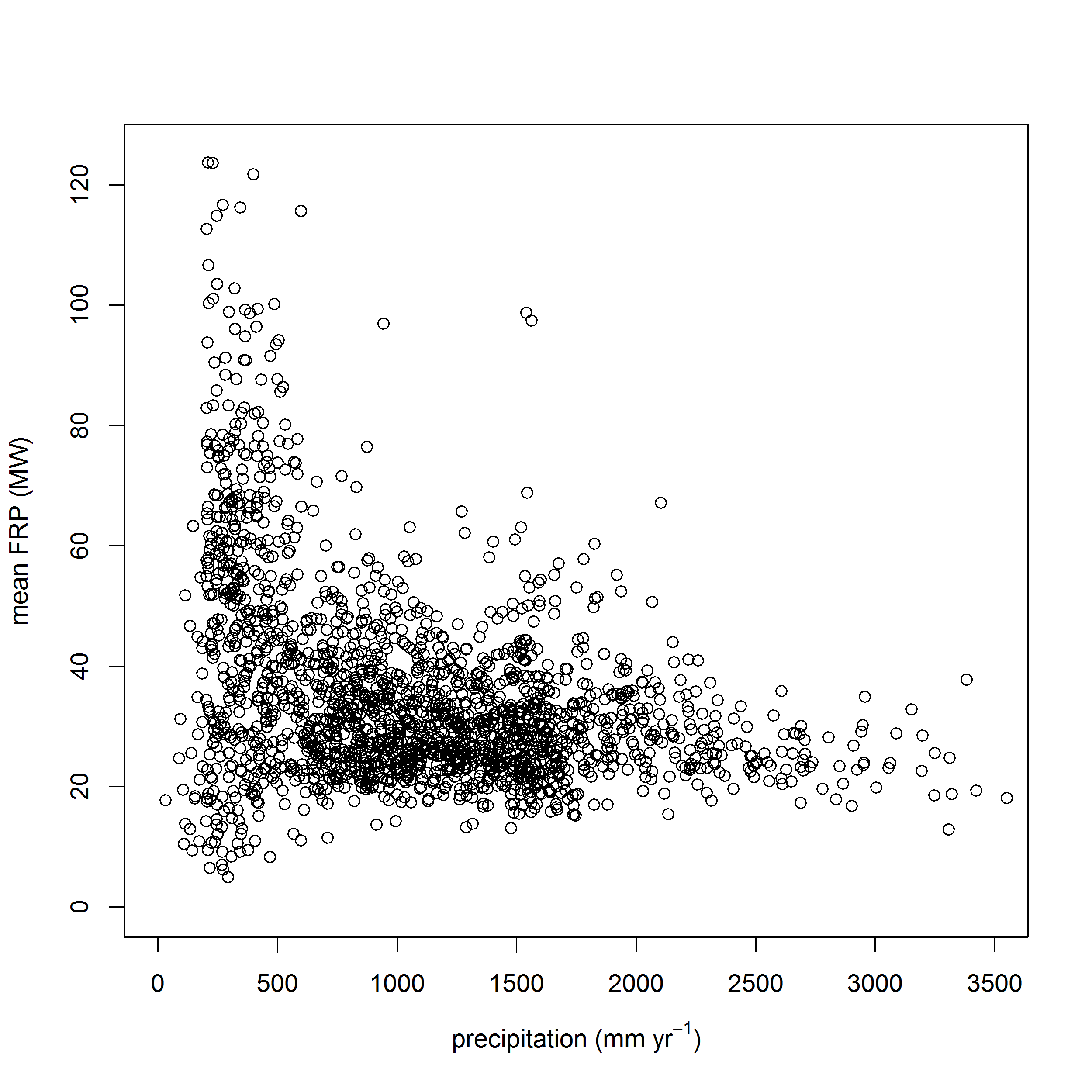


Figure S9


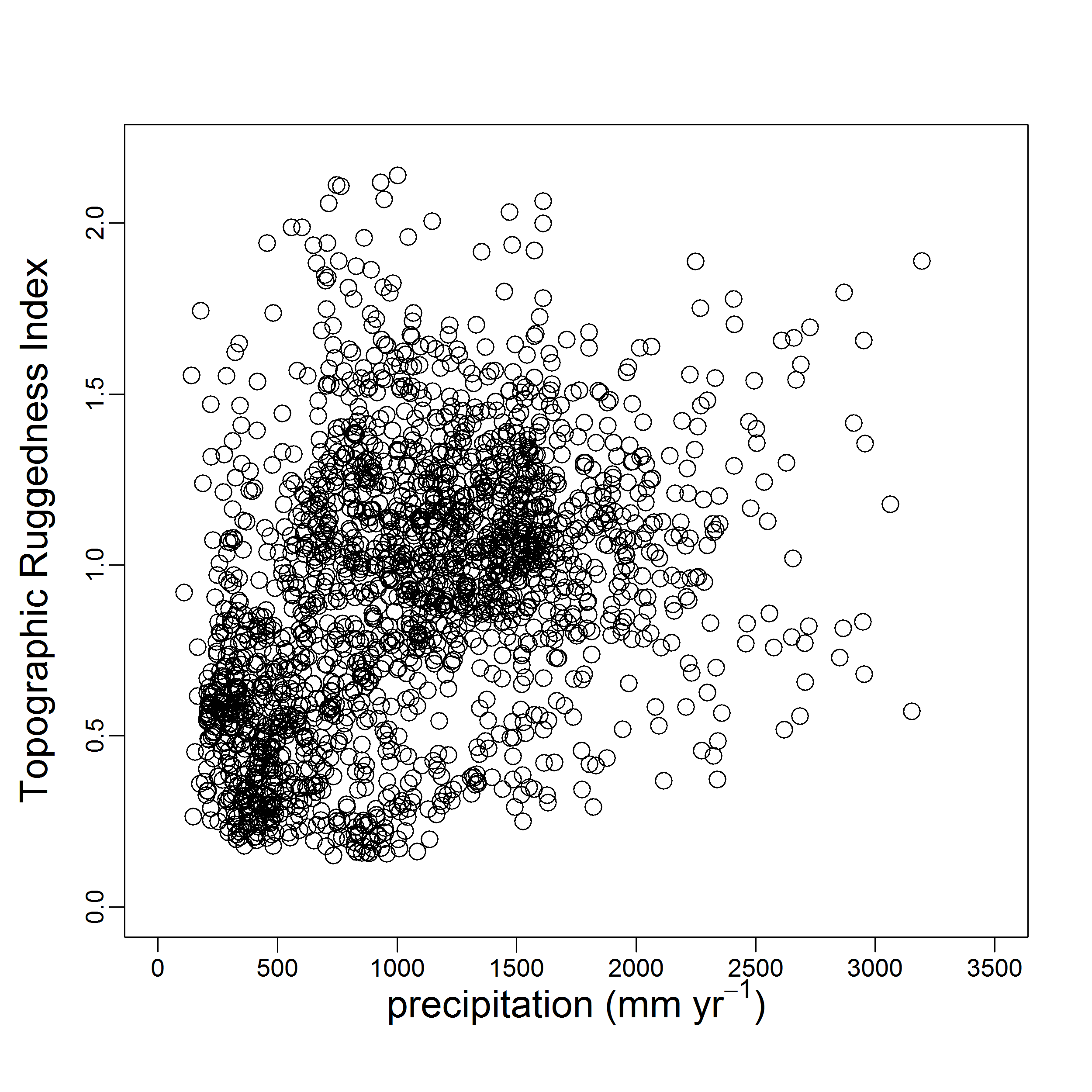


Figure S10


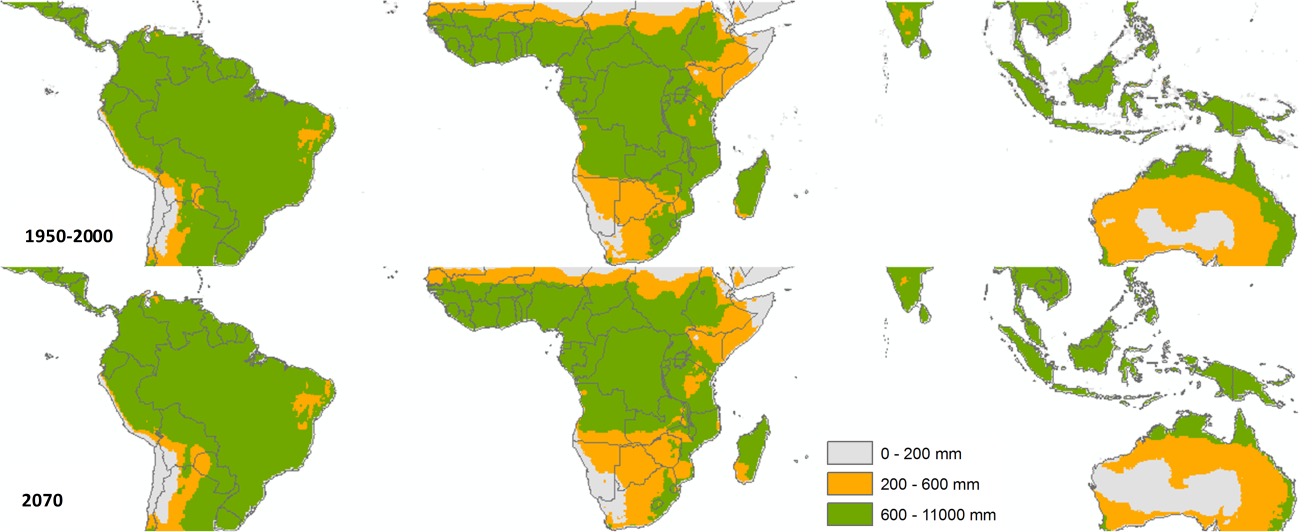


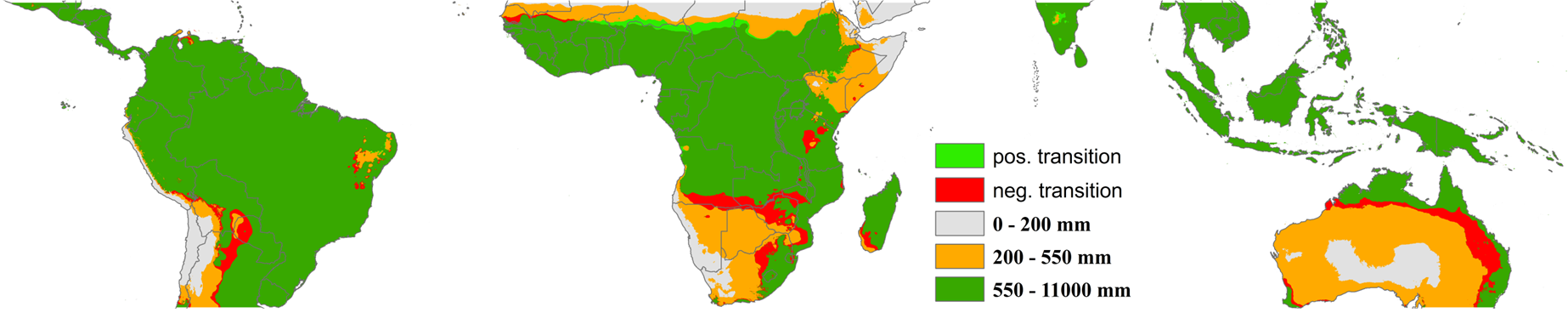

Supplement: Supplementary file 1 — supplementary information [file 41598_2017_14654_MOESM1_ESM.doc]
